# Mental health problems in pregnant and postpartum women living with HIV in sub-Saharan Africa: Systematic review and meta-analysis protocol

**DOI:** 10.1371/journal.pone.0308810

**Published:** 2024-10-03

**Authors:** Anthony Danso-Appiah, Kwadwo Owusu Akuffo, David Owiredu

**Affiliations:** 1 Centre for Evidence Synthesis and Policy, University of Ghana, Legon, Accra, Ghana; 2 Department of Epidemiology and Disease Control, School of Public Health, University of Ghana, Legon, Accra, Ghana; 3 Africa Communities of Evidence Synthesis and Translation (ACEST), Accra, Ghana; 4 Department of Optometry and Visual Science, College of Science, Kwame Nkrumah University of Science and Technology, Kumasi, Ghana; University of Connecticut Health Center: UConn Health, UNITED STATES OF AMERICA

## Abstract

**Background:**

Existing evidence on the burden of mental health problems among pregnant and postpartum women living with HIV, a vulnerable population in sub-Saharan Africa (SSA), is limited and fragmented, affecting the development of context-sensitive and integrated interventions. This systematic review aims to provide an up-to-date and comprehensive synthesis of available evidence to estimate the burden and identify the determinants of mental health problems among pregnant and postpartum women living with HIV across countries in sub-Saharan Africa.

**Methods:**

We will retrieve all relevant studies (published and unpublished) through searches in PubMed, Embase, PsycINFO, CINAHL, LILACS, Google Scholar, Scopus and Web of Science from inception to 30^th^ June 2024, without language restriction. We will use the following search terms ‘mental health disorder’, ‘mental health problem’, ‘pregnant women’, ‘postpartum women’ and ‘HIV’ nested with all applicable alternate terms and the names of countries in SSA for running the searches. We will also search HINARI, African Index Medicus, African Journals Online, Academic Search Premier, medRxiv, ProQuest, EBSCO Open Dissertations, and reference lists of relevant studies. We will contact experts in the field for potentially relevant unpublished studies. All retrieved articles from the electronic databases and grey literature will be collated and deduplicated using Endnote and exported to Rayyan QCRI. Two reviewers will independently select studies using a pretested study selection flow chart developed from the pre-specified eligibility criteria. Two reviewers will extract data using a pretested data extraction form and assess the risk of bias in the included studies using the risk of bias tool for prevalence studies by Hoy et al. (2012). Any disagreements will be resolved through discussion between the reviewers. Binary outcomes (prevalence and incidence of mental health problems among pregnant and postpartum women living with HIV) will be evaluated using pooled proportions (for non-comparative studies) and odds ratio (OR) or risk ratio (RR) (for comparative studies), and mean difference for continuous outcomes, all will be reported with their 95% confidence intervals (CIs). Heterogeneity will be assessed graphically for overlapping CIs and statistically using the I^2^ statistic. If substantial heterogeneity is found, random-effects model meta-analysis will be performed; otherwise, fixed-effect meta-analysis will be employed. We will conduct subgroup analysis (to assess the impact of heterogeneity) and sensitivity analyses to test the robustness of the generated effect estimates to the quality domains. The overall level of evidence will be assessed using GRADE (Grading of Recommendations, Assessment, Development, and Evaluations).

**Expected outcomes:**

The review is expected to produce an up-to-date and comprehensive synthesis of the available evidence, allowing for the generation of country-specific estimates of the burden of mental health problems among mothers living with HIV across SSA populations. Also, the review will attempt to identify the determinants of mental health problems among pregnant and postpartum women living with HIV, to shed light on the factors that contribute to the occurrence of mental health problems in this vulnerable population.

**Systematic review registration:**

The systematic review protocol has been registered in the International Prospective Register for Systematic Reviews (PROSPERO), with registration ID CRD42023468537.

## Background

Mental health problems in pregnant and postpartum women are increasingly gaining attention worldwide due to their impact on the well-being of the mother, foetus and infant, as well as families and communities [1, 2]. Globally, mental health disorders affect a high proportion of women during pregnancy and in the postpartum period [3–6]. Approximately, 10% of pregnant women and 13% of postpartum women experience one of the common mental disorders [1], primarily due to their physiological and hormonal changes [5, 7]. Human Immunodeficiency virus (HIV) infection disproportionately affects women [8–10], with pregnant and postpartum women being at a higher risk due to immune and hormonal alterations and shifts in the vaginal microbiome [11, 12].

Mental health problems among pregnant and postpartum women are responsible for increased morbidity and mortality among childbearing women worldwide, with stark disparities between high-income countries (HICs), low and middle-income countries (LMICs) and SSA [13–15]. For example, in the United States of America, the United Kingdom, and mainland Europe, approximately 1 in 5 (20%) of women reportedly experience a mental health problem during pregnancy and the postpartum period [16, 17]. Systematic reviews focusing on individual countries [18, 19] or regional [20, 21] and global contexts [22] have all consistently reported similar findings. The proportions of maternal mental health problems are higher for LMICs and SSA [14, 23–25], with some studies reporting rates as high as 39% for pregnant women [26] and 50% for postpartum women [27]. It has been suggested that limited access to healthcare services [23, 28, 29], poverty [30, 31], gender inequality [1, 31, 32], and conflicts [13, 14, 33, 34] are key factors affecting maternal mental health problems, whereas stigma [34, 35] and lack of awareness of mental health issues [6, 36] hinder proper care for mental health issues in SSA [37, 38]. Teenage pregnancy, which is associated with poor psychosocial support and an increased risk of depression if not detected early and treated, disproportionately affects teenage girls living in SSA [39, 40].

Globally, HIV continues to pose a significant public health threat, with women accounting for a high proportion of those affected [8]. Compared to other global sub-regions, SSA has the highest burden of HIV infections, accounting for over 66% of the global estimate in 2022 [8]. In that same year, 63% of all new HIV infections in the sub-region occurred in women and girls [8]. Similar to mental health problems, the high proportion of HIV cases in SSA has been attributed to poverty [41–43], gender inequality [8, 44], cultural practices [45, 46], low literacy levels, and inadequate access to healthcare services [43, 47], among others. Worryingly, an estimated 1.3 million women and young girls living with HIV become pregnant annually [9], further putting them at risk of a host of health problems, including those that affect their psychological well-being.

Mental health problems in pregnant and postpartum women living with HIV present complex challenges to the general well-being, care, and health outcomes of both the mother and baby [48–51]. The introduction of antiretroviral therapy (ART) has resulted in a reduction in viral load, HIV-related morbidities, and mortalities [52], and increasingly, more women living with HIV in childbearing age are seeking to become pregnant [53, 54]. However, pregnant and postpartum women living with HIV usually face a unique set of stressors and vulnerabilities [51], including heightened levels of anxiety [7], depression [55], and emotional distress [56] that may stem from their awareness of the risk of transmission of the infection to the foetus during pregnancy or the infant through breastfeeding [57]. HIV-associated stigmatisation may also further exacerbate the already compromised mental state of pregnant and postpartum women living with HIV [55–59]. On the other hand, the presence of maternal mental health problems may impact adherence to ART [60] and participation in prenatal and postnatal care [58, 61].

### Rationale for this systematic review

Despite the reportedly high burden of mental health problems in pregnant and postpartum women living with HIV in SSA [1, 8, 14, 15], no systematic review has comprehensively generated robust country-specific estimates of the burden, risk factors, quality of life, interventions, and pregnancy outcomes related to mental health problems in this vulnerable population [14, 15, 62, 63]. This knowledge gap hinders the development of targeted and evidence-based interventions to address the mental health needs of this vulnerable population living with HIV. Existing systematic reviews of mental health problems usually focus on the general population and are often restricted to selected conditions, mainly depression and anxiety [64–67]. From comprehensive searches conducted as part of the preparation of this protocol, no systematic review of mental health problems among pregnant and postpartum women living with HIV in SSA exists. The only systematic review that sought to collate evidence on common mental health disorders among adolescents living with HIV in SSA [15] involved just one study [68] that met their inclusion criteria, suggesting a serious dearth of literature and evidence. Another systematic review investigating evidence on perinatal depression in HIV-infected African women conducted over a decade ago in 2014 [69], is not only outdated but concentrated on depression. A scoping review investigated the general experiences of adolescent mothers affected by HIV (not necessarily having HIV themselves) and their children but the focus was not mental health [70]. The present review seeks to provide an up-to-date and comprehensive synthesis of the available evidence, estimate country-specific burden and determinants of mental health problems among pregnant and postpartum women living with HIV, and assess the maternal and foetal birth outcomes among this vulnerable population across countries in SSA.

By collating evidence on mental health problems in this key population living with HIV, the review will contribute to promoting good health and wellbeing, a central objective of Sustainable Development Goal Three (SDG 3); by addressing sub-targets 3.1, 3.3, 3.4, and 3.5—to reduce maternal mortality and non-communicable diseases, and promote the general mental wellbeing. In addition, the review acknowledges the unique challenges faced by women living with HIV, highlighting the intersection of gender inequality and mental health. By targeting these issues, it lends to SDG 5- Gender equality and contributes to the promotion of gender equality and empowerment of women in the SSA region.

## Methods

The current review protocol has been prepared following the Preferred Reporting Items for Systematic Review and Meta-Analysis extension for protocols (PRISMA-P) checklist [71] (S1 Table) and as the full review report will be guided by the PRISMA checklist [72]. We have also followed the guidance specified in the Cochrane Handbook [73] and methods reported in earlier works [74–83] and used the PECOS framework to describe the Population, Exposure, Comparator, Outcomes, and Study types. The databases and other sources searched, and the flow of studies will be reported using the PRISMA Flow Diagram [72] (S1 Fig).

### Patient and public involvement

The review questions and outcomes to be assessed have been developed collaboratively with patients and public involvement, and informed by their priorities, experiences and preferences in line with GRIPP2 reporting checklists [84]. The review findings will be shared with relevant patient communities who will also be involved in the dissemination of the results.

### Ethical considerations and dissemination

This is secondary research based on published studies or publicly available data. Therefore, no ethical approval is required. However, an eligible study with serious ethical issues will be excluded, and the reasons for exclusion documented. Study findings will be presented at conferences, and copies will be shared with relevant stakeholders, health authorities, and agencies involved in the mental health of mothers who are living with HIV. The final review report will be in the form of a scientific paper for submission for publication in a high-impact factor peer-reviewed journal.

### Criteria for including studies for this review

#### Type of studies

Any study (cohort, case-control, and cross-sectional design) conducted in a country in sub-Saharan Africa that assessed maternal mental health conditions in pregnant and postpartum women living with HIV will be eligible for inclusion. Randomized controlled trials (RCTs) reporting baseline prevalence of maternal mental health disorders in a well-defined sample of pregnant or postpartum women living with HIV will be considered for inclusion. Literature reviews, scoping reviews, and systematic reviews will not be included. However, the list of included/reviewed studies (especially from any retrieved global systematic review on the subject) will be perused for sub-Saharan African studies for inclusion in this review. Case studies, case series (are atypical and do not represent the source population), opinions, commentaries, and editorial letters will be excluded.

#### Population

Pregnant and postpartum women living with a confirmed diagnosis of HIV and mental health conditions, including anxiety disorder, depression, psychosis, schizophrenia, suicidal ideation, paranoia, substance use disorder, Obsessive Compulsive Disorder, Posttraumatic Stress Disorder, Self-harm, etc., will be eligible for inclusion in the review. The tool or criteria used for diagnosing HIV (ELIZA, PCR, etc.) and/or mental health conditions such as the Diagnostic Statistical Manual of Mental Disorders (DSM-V) [85], the Depression, Anxiety and Stress Scale-21 Items (DASS-21) [86], the Edinburgh Postnatal Depression Scale (EPDS) [87] and Self Reporting Questionnaire-20 items (SRQ-20) [88] must be reported or documented. The tools for diagnosing the HIV infection as specified in the exposure should be documented. Pregnant or postpartum women living with HIV whose diagnosis of a mental health disorder could not be confirmed will be excluded. The study should involve participants resident in any of the sub-Saharan African countries to be eligible for inclusion. Studies involving sub-Saharan African nationals living outside SSA will be excluded.

#### Exposure

A clinically confirmed (and documented) diagnosis of HIV using any standard testing algorithms, including initial screening with, for example, the enzyme-linked immunosorbent assay (ELISA) [89] and confirmed by polymerase chain reaction (PCR) [90]. All confirmed cases will be considered eligible participants (exposed) irrespective of the stage of HIV infection (whether acute or chronic HIV or acquired immunodeficiency syndrome (AIDS)).

#### Comparators

This is not a comparative review, but where necessary, subgroup comparisons will be made.

#### Outcomes

Primary outcomes.

1. Prevalence and incidence of mental health disorders among pregnant and postpartum women living with HIV in SSA
2. Birth outcomes (such as livebirths, stillbirths, preterm births, low birth weight, obstetric complications) of HIV-positive women with mental health disorders

Secondary outcomes.

1. Sociodemographic determinants of mental health disorders in pregnant and postpartum women living with HIV in SSA
2. Mental health predisposition of pregnant and postpartum women living with HIV
3. Obstetric predisposition of pregnant and postpartum women living with HIV having mental health problems
4. Medical predisposition of pregnant and postpartum women living with HIV to having mental health problems

### Search methods for identification of studies

#### Electronic databases

We will retrieve all relevant studies (published and unpublished) through searches in PubMed, Embase, PsycINFO, CINAHL, LILACS, Google Scholar, Scopus and Web of Science from inception to 30^th^ June 2024, without language restriction. We will use the following search terms: ‘mental health disorder,’ ‘mental health problem’ ‘pregnant women,’ ‘postpartum women,’ and ‘HIV’ nested with all applicable alternate terms and the names of countries in SSA for running the searches (see Table 1 for further details) to give a comprehensive search strategy. The names of all the 48 countries in sub-Saharan Africa (according to the World Bank records) [91] will also be added as alternative search terms. The searches will be extended to HINARI, African Index Medicus, African Journals Online, Academic Search Premier, preprint databases (for example, medRxiv), grey literature sources (including conference proceedings) and dissertation/theses (ProQuest and EBSCO Open Dissertations), and specialised data repositories. A detailed search strategy developed for PubMed will be adapted for the searches in the other databases.

**Table 1:** Search strategy developed for PubMed, to be adapted for the other databases.

Search	Query
**#1**	Search: ((((((((((((((((((((((((("mental health"[Title/Abstract]) OR ("mental health problem*"[Title/Abstract])) OR ("psychological disorder*"[Title/Abstract])) OR ("emotional disorder*"[Title/Abstract])) OR ("mental disorder*"[Title/Abstract])) OR ("mental illness*"[Title/Abstract])) OR ("psychiatric illness*"[Title/Abstract])) OR ("psychiatric disorder*"[Title/Abstract])) OR (depression[Title/Abstract])) OR ("depressive disorder*"[Title/Abstract])) OR (anxiety[Title/Abstract])) OR (bipolar[Title/Abstract])) OR ("bipolar affective disorder*"[Title/Abstract])) OR (paranoia[Title/Abstract])) OR (psychopathy[Title/Abstract])) OR (neuros*[Title/Abstract])) OR ("neurotic disorder*"[Title/Abstract])) OR (schizophrenia[Title/Abstract])) OR ("schizophrenic disorder*"[Title/Abstract])) OR (suicide[Title/Abstract])) OR (suicidal[Title/Abstract])) OR ("suicidal ideation"[Title/Abstract])) OR (self-harm[Title/Abstract])) OR (infanticide[Title/Abstract])) OR ("alcohol dependence"[Title/Abstract])) OR ("substance use"[Title/Abstract])
**#2**	Search: ((((((((((((("pregnant women"[Title/Abstract]) OR (prepartum[Title/Abstract])) OR (peripartum[Title/Abstract])) OR (perinatal[Title/Abstract])) OR (prenatal[Title/Abstract])) OR (antenatal[Title/Abstract])) OR ("during pregnancy"[Title/Abstract])) OR (postpartum[Title/Abstract])) OR (maternal[Title/Abstract])) OR (maternity[Title/Abstract])) OR (parturient[Title/Abstract])) OR (antepartum[Title/Abstract])) OR ("post-delivery"[Title/Abstract])) OR (puerperium[Title/Abstract])
**#3**	Search: ((((((((HIV[MeSH Terms]) OR (HIV/AIDS[MeSH Terms])) OR (“human immunodeficiency virus” [MeSH Terms])) OR (“Acquired Immune deficiency syndrome” [MeSH Terms])) OR (AIDS[MeSH Terms])) OR (“people living with HIV” [Title/Abstract])) OR (PLHIV[Title/Abstract])) OR (“people living with HIV/AIDS” [Title/Abstract])) OR (PLWHA[Title/Abstract])
**#4**	Search: (((((((((((((((((((((((((((((((((((((((((((((((((“sub-Saharan Africa”) OR (SSA)) OR (Angola)) OR (Benin)) OR (Botswana)) OR (“Burkina Faso”)) OR (Burundi)) OR (Cameroon)) OR (“Cape Verde”)) OR (“Central African Republic”)) OR (Chad)) OR (Comoros)) OR (Congo)) OR (“Cote d’Ivoire”)) OR (Djibouti)) OR (“Equatorial Guinea”)) OR (Ethiopia)) OR (Gabon)) OR (“The Gambia”)) OR (Ghana)) OR (Guinea)) OR (“Guinea-Bissau”)) OR (Kenya)) OR (Lesotho)) OR (Liberia)) OR (Madagascar)) OR (Malawi)) OR (Mali)) OR (Mauritania)) OR (Mauritius)) OR (Mozambique)) OR (Namibia)) OR (Niger)) OR (Nigeria)) OR (Rwanda)) OR (“Sao Tome and Principe”)) OR (Senegal)) OR (Seychelles)) OR (“Sierra Leone”)) OR (Somalia)) OR (“South Africa”)) OR (Sudan)) OR (Swaziland)) OR (Tanzania)) OR (Togo)) OR (Uganda)) OR (Zaire)) OR (Zambia)) OR (Zimbabwe)) OR (Africa)
**#5**	Search: (#1) AND (#2)
**#6**	Search: (#3) AND (#5)
**#7**	Search: (#4) AND (#6)

#### Other sources

The reference lists of all relevant studies (including systematic, scoping, and literature reviews) will be hand-searched to identify potentially relevant studies. For global systematic reviews where sub-Saharan Africa has been considered a subgroup, the included studies will be reviewed, and those missed by our searches will be retrieved for assessment and inclusion. Experts in the field will also be contacted via the most appropriate means (emails, WhatsApp, etc.) for any knowledge about potentially relevant publications missed by our searches and unpublished studies.

### Screening and selection of studies

**Fig 1:**
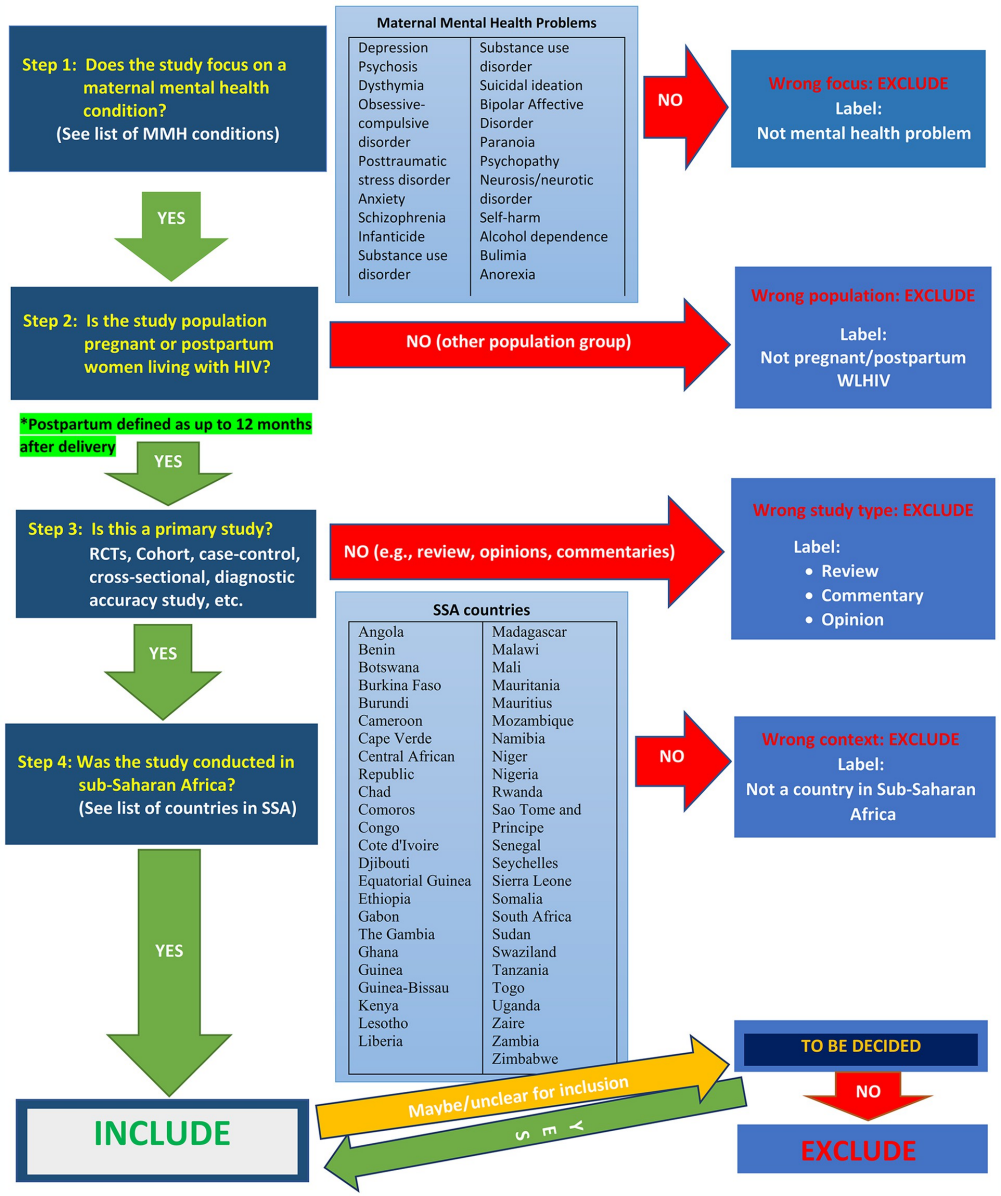
Study selection flow chart developed from the pre-defined eligibility criteria.

All the articles retrieved from the searches will be collated and deduplicated using EndNote. The deduplicated studies will then be exported to Rayyan (a web-based systematic review application) [92], where studies will be screened and selected. Two reviewers (DO and KOA) will independently screen studies using a study selection flow chart developed from the pre-specified eligibility criteria for considering studies for inclusion in the review (Fig 1), using a two-phase approach. First, the titles and abstracts of all retrieved studies will be screened to exclude apparent irrelevant studies using the pretested study selection flow chart. In the second phase, full texts of potentially relevant studies will be retrieved and subjected to further screening, and those that meet the inclusion criteria will be selected for inclusion in the review. Conflicts will be resolved through discussion between the reviewers. The study selection process and the respective decisions for inclusion and exclusion of studies will be summarised and presented using the PRISMA flow diagram (S1 Fig) [93]. All studies excluded at the full-text stage will be listed in a table, and the reason(s) for the exclusion provided.

### Data extraction and management

Relevant data from the included studies will be extracted independently by at least two reviewers (ADA, DO and KOA) and transferred to a Microsoft Excel [94] spreadsheet. The extraction process will be pretested using randomly selected studies to ensure consistency between data extractors. Study characteristics, such as the year of publication (for published studies), year study was conducted, the country where the study was conducted, study design, type of participants (pregnant and postpartum women), sample size; sociodemographic characteristics (age of participants, level of education, residence (rural or urban), occupation, occupation, employment status etc.); obstetric characteristics (parity, gravidity, age of first delivery, history of obstetric events such as miscarriage and stillbirth); and other clinical characteristics (such as HIV viral load counts, viral suppression status, ART enrolment status and the presence of other co-morbid conditions) will be extracted. Data on mental health conditions and diagnostic tools used will also be extracted from each included study. In studies where the primary estimates are not reported and do not have the minimum information to calculate the relevant estimates, the authors will be contacted directly via email, WhatsApp or phone call, where feasible, to provide the required information. In situations where it is not possible to obtain the missing data from the primary authors and the amount and reasons for the missing data will be reported. If the data from multinational studies have been lumped together, we will attempt to disaggregate the data by country. Where it is impossible to do disaggregate the data, such studies will be presented as one, accompanied with citations of the included countries. The extracted data will be verified independently, and any conflict will be resolved through discussion.

### Assessment of quality of the included studies

The Risk of Bias Tool for Prevalence Studies [95] (S2 Table) will be used to evaluate the quality of the included observational studies for risk of bias on participant selection, non-response, and bias arising from measurement and data analysis methods. A determination of “low risk” of bias, “high risk” of bias, or “unclear” bias will be made for each bias domain accompanied by the assessor’s supporting information. The risk of bias assessment will be carried out by one reviewer (DO) and independently verified by ADA. Any disagreement will be resolved through discussions between the assessors.

### Data analysis

The cleaned data will be analysed with Review Manager (RevMan)-Version 7.2.0) [96] for the data analysis, and where necessary, analyse some of the data in Stata-Version 15 [97]. Binary outcomes will be analysed and presented as odds ratio (OR) or risk ratio (RR), and for continuous data, we will employ mean difference (MD) or standardized mean difference (SMD) where different scales have been used. To compute the weighted average of the prevalence or incidence of maternal mental health disorders across studies, meta-analyses will be conducted to distil evidence at the highest possible level. The individual study’s OR, RR, and MD will be pooled and presented along with their corresponding 95% confidence intervals (CI). In instances of high heterogeneity among studies, we will adopt a random-effects model for the meta-analysis; otherwise, a fixed-effect model will be used. Additionally, descriptive statistics will be used to present proportions. Forest plots will be used to demonstrate the summary estimates with their CIs and weighted contributions to the pooled estimate. For trend-related outcomes, Bayesian analysis will be conducted through the use of meta-regression to summarize the effect estimates.

### Assessment of heterogeneity and subgroup analyses

Heterogeneity will be assessed visually by inspecting overlaying CIs of the forest plots and outlying data and quantitatively using the I^2^ statistic. The variations in the studies included in the meta-analysis will be assessed by the I^2^-statistic, a measure of variation across studies due to heterogeneity rather than chance [98]. The I^2^ values will be classified as ≤ 25% (low), 50% (moderate), 75% (high), and ≥ 75% (significant) heterogeneity [99]. Where significant heterogeneity is detected, a subgroup analysis will be performed to detect the possible sources of variation, for example, study design, study setting, type of mental health disorder, parity, HIV viral suppression status, and geographic location (Central, Eastern, Southern and Western Africa).

### Sensitivity analyses

Sensitivity analyses [100] will be performed to assess the impact of outliers in the included studies, particularly on the quality domains (for example, studies with large sample sizes, high prevalence or incidences, very precise confidence intervals, or very high risk of bias of any of the quality domains) on the resultant estimates. Meta-analyses will be performed, first with and then without such studies, to assess the robustness of the effect estimates. The impact of missing data and unpublished studies will also be adequately evaluated in sensitivity analysis to test the robustness of the pooled estimates.

### Dealing with missing data

The pattern of missing data and the potential impact on the study findings and conclusions, as well as the reasons for the missing data will be explored. We will not employ imputation as a means of addressing missing data, but instead, we will contact primary study authors and ask for the missing data or, where possible, ask for the raw data to enable us to extract the missing information.

### Assessing the certainty of evidence using GRADE

The Grading of Recommendations Assessment, Development and Evaluation (GRADE) approach [101] will be used to assess the overall quality or certainty of the evidence (how certain the authors are that the effect estimate represents the true effect) on the key outcomes provided by the included studies. Two reviewers (ADA and DO) will assess the certainty of evidence (*quality* of evidence or the *confidence* in the effect estimates) from the following five domains: risk of bias, inconsistency, indirectness, imprecision, or publication bias. The certainty of evidence will be graded as high, moderate, low, or very low. The certainty of evidence can be rated down by one or two levels when there are serious or very serious concerns, respectively, in any of the domains [101, 102]. The certainty of evidence upward for high-quality observational studies, although this is usually rarely done [102, 103]. An overall rating of the certainty of the evidence for each outcome will be presented in a summary of findings table together with the study types, the number of studies and participants, and the relative and absolute effects for each outcome [103].

## Discussion

This systematic review and meta-analysis protocol is driven by the urgent need to address the notable gap in the literature regarding mental health problems among pregnant and postpartum women living with HIV in sub-Saharan Africa. Despite the increasing recognition of the intersection between mental health, HIV/AIDS, and maternal health, comprehensive evidence on the prevalence, determinants, interventions, and outcomes related to mental health issues in this vulnerable population remains deficient. This protocol aims to address this void by providing a detailed plan for synthesizing existing data and generating unbiased evidence regarding mental health problems among pregnant and postpartum women living with HIV in SSA.

In this systematic review and meta-analysis protocol, a detailed plan of all the steps of the review, including eligibility criteria, information sources, search strategy, study selection, data extraction, quality assessment, and data synthesis, has been described. This meticulous approach ensures errors and biases are minimised. By adhering to rigorous methodology and transparent reporting practices, we aim to provide reliable findings on the incidence, prevalence, determinants, and outcomes of mental health problems in this vulnerable population. Ultimately, the knowledge obtained from this review will serve as a valuable resource for clinicians, policymakers, and researchers working to improve the mental health and overall well-being of pregnant and postpartum women living with HIV in sub-Saharan Africa.

### Strengths and limitations

This systematic review and meta-analysis will attempt to collate and distil empirical evidence at the highest level using explicit, transparent, and reproducible methods to answer the review question, ensuring strict adherence to evidence synthesis best practices. The process will involve a comprehensive search strategy operationalized on multiple databases and non-database sources to attempt to retrieve all potentially relevant studies. The selection of studies, data extraction, and assessment of quality of the included studies will be guided by validated or piloted instruments. It will be done in duplicates to minimize errors and biases. We will run updated searches until the final analyses start. A key limitation could be the inability to access complete data from some relevant studies, particularly very old studies, given that we have not placed any restrictions on the year was conducted or published. However, this is not expected to influence the magnitude and direction of the effect estimates since it is anticipated that the large number of studies will meet the inclusion criteria. Furthermore, completeness, validity, and reliability of the evidence generated are not expected to be severe issues given the rigorous methods and the review process itself employed, which will be guided by best practices.

### Implications of the anticipated review findings

The findings of this review will support the development of targeted interventions and policies aimed at addressing the underlying drivers of poor mental health outcomes among pregnant and postpartum women living with HIV. Our study protocol anticipates highlighting the evidence on the impact of mental health problems on maternal and foetal outcomes among pregnant women living with HIV in sub-Saharan Africa. By synthesising data on outcomes such as maternal mortality, infant mortality, and perinatal complications among pregnant and postpartum women living with HIV, we aim to elucidate the downstream consequences of untreated mental health issues in this population. This understanding is essential for informing clinical practice and public health interventions aimed at improving maternal and foetal health outcomes in the context of HIV/AIDS.

## Supporting information

S1 Fig PRISMA 2020 flow diagram for new systematic reviews which included searches of databases, registers and other sources. (TIF)

S1 Table PRISMA-P (Preferred Reporting Items for Systematic Review and Meta-Analysis Protocols) 2015 checklist: Recommended items to address in a systematic review protocol. (DOCX)

S2 Table Quality assessment checklist for prevalence studies (adapted from Hoy et al. 2012). (DOCX)
