# Supplementary figures and images for: Mental health problems in pregnant and postpartum women living with HIV in sub-Saharan Africa: Systematic review and meta-analysis protocol
Source: PLoS One. 2024 Oct 3;19(10):e0308810. doi: 10.1371/journal.pone.0308810 (PMC11449370; doi:10.1371/journal.pone.0308810)

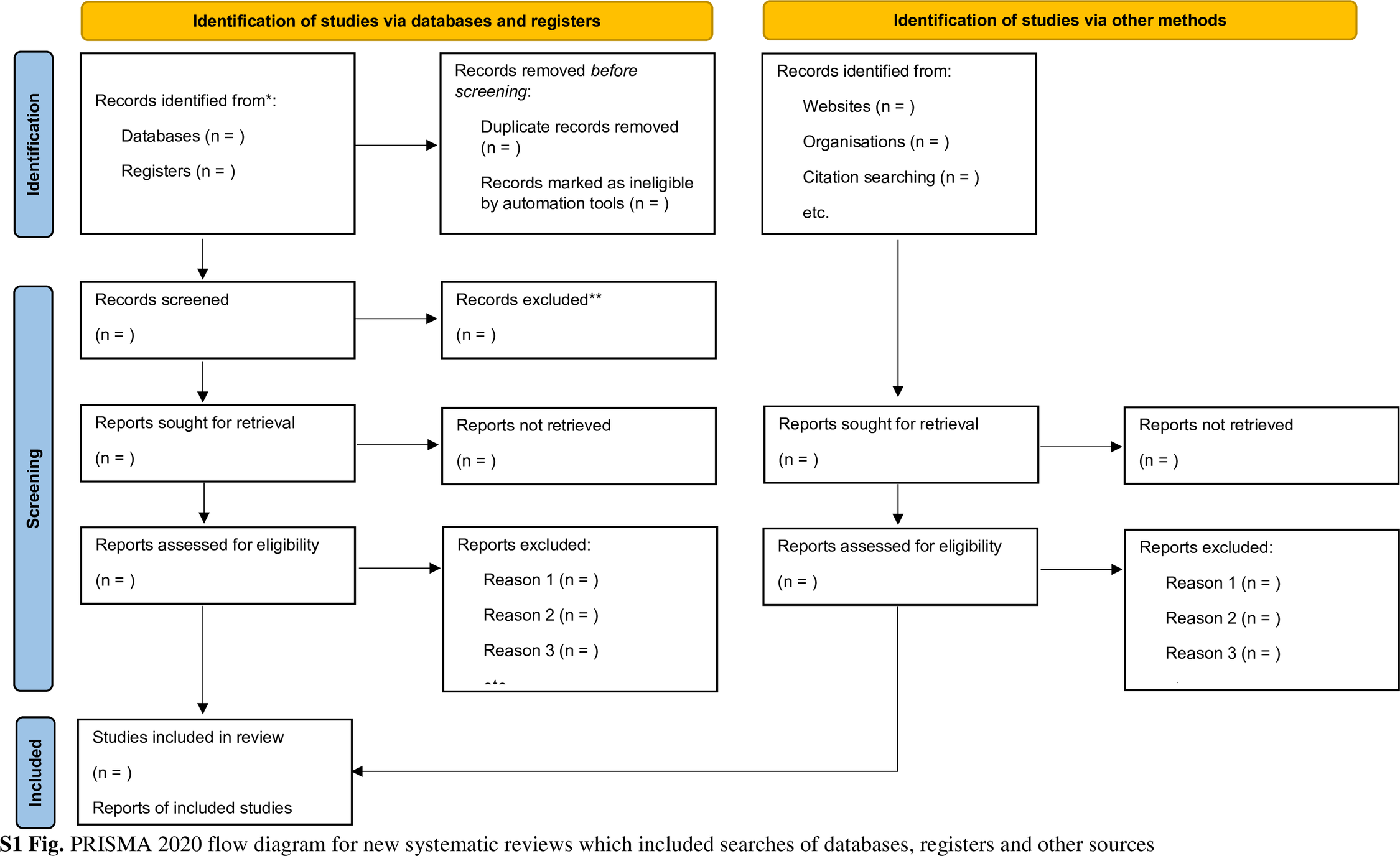

Supplement: S1 Fig — (TIF) [file pone.0308810.s001.tif]
